# Dietary total antioxidant capacity is inversely associated with the odds of non-alcoholic fatty liver disease in people with type-2 diabetes

**DOI:** 10.3389/fnut.2022.1037851

**Published:** 2022-11-03

**Authors:** Marieh Salavatizadeh, Samira Soltanieh, Hossein Poustchi, Zahra Yari, Maryam Shabanpur, Asieh Mansour, Mohammad E. Khamseh, Fariba Alaei-Shahmiri, Azita Hekmatdoost

**Affiliations:** ^1^Department of Clinical Nutrition and Dietetics, Faculty of Nutrition and Food Technology, Shahid Beheshti University of Medical Sciences, Tehran, Iran; ^2^Endocrine Research Center, Institute of Endocrinology and Metabolism, Iran University of Medical Sciences, Tehran, Iran; ^3^Liver and Pancreatobiliary Diseases Research Center, Digestive Diseases Research Institute, Shariati Hospital, Tehran University of Medical Sciences, Tehran, Iran; ^4^Department of Nutrition Research, Faculty of Nutrition Sciences and Food Technology, National Nutrition and Food Technology Research Institute, Shahid Beheshti University of Medical Sciences, Tehran, Iran; ^5^Department of Nutrition, Jahrom University of Medical Sciences, Jahrom, Iran; ^6^Endocrinology and Metabolism Research Center, Endocrinology and Metabolism Clinical Sciences Institute, Tehran University of Medical Sciences, Tehran, Iran

**Keywords:** NAFLD, diabetes, diet, DTAC, antioxidant

## Abstract

**Background:**

This study was conducted to evaluate possible associations between Dietary Total Antioxidant Capacity (DTAC) and odds of non-alcoholic fatty liver disease (NAFLD) in people with type-2 diabetes mellitus (T2DM).

**Materials and methods:**

We recruited two hundred people with T2DM, and evaluated their liver steatosis using Fibroscan. Dietary intakes of participants were assessed using a validated food frequency questionnaire. DTAC was computed *via* ferric reducing antioxidant power (FRAP).

**Results:**

In the crude model, no statistically significant association was found between DTAC and the odds of NAFLD in people with diabetes. However, after adjustment for potential confounders including age, gender, diabetes duration, smoking status, physical activity, BMI, waist circumference, and energy, the most reduced adjusted OR was indicated for the third tertile *vs.* the first one (OR: 0.28, 95% CI: 0.09–0.81, *P* = 0.02), meaning that diabetic patients in the third tertile of DTAC had 72% decreased risk of NAFLD in comparison to those in the first one. The relationship was remained significant after additional adjustment for HOMA-IR, HbA1c, serum Triglyceride (TG), and low-density lipoprotein-cholesterol (LDL) levels (OR: 0.29, 95% CI: 0.09–0.93, *P* = 0.03). Importantly, a dose-response pattern was demonstrated for DTAC and risk of NAFLD (*P* = 0.04).

**Conclusion:**

Higher DTAC was related with a decreased risk of NAFLD in individuals with diabetes.

## Introduction

Non-alcoholic fatty liver disease (NAFLD), an obesity complication commonly overlooked, is defined by the deposition of more than 5% fat in the liver not resulting from other identifiable factors such as alcohol consumption or viral hepatitis, ranging from hepatic steatosis to fibrosis and related cirrhosis (1, 2). Non-alcoholic steatohepatitis (NASH) is a more severe form of NAFLD featured by inflammation, hepatocyte necrosis, and regularly fibrosis (3). As a major cause of liver disease globally, NAFLD and NASH give rise to a substantial burden (4). A recent meta-analysis of 86 studies in 2016, estimated the prevalence of NAFLD in general population worldwide at 25.24%, whereas several meta-analyses demonstrated that in 2017 the prevalence was twofold (54–59.67%) in people with type 2 diabetes mellitus (T2DM) (2, 5, 6). The link between NAFLD and T2DM has been clearly identified, and can be described by IR and hyperinsulinemia resulting in defective lipid metabolism and the accumulation of fatty acids in the liver (7). Moreover, in people suffering from T2DM and NASH, the process of oxidative stress is increased, compared to T2DM patients without NAFLD (8, 9). T2DM furthermore enhances the susceptibility to advanced NAFLD including NASH, liver fibrosis, and hepatocellular carcinoma (4). Therefore, it seems vital to identify and monitor these high-risk patients.

The gold standard procedure for distinguishing NAFLD is liver biopsy, however, is impossible to apply an invasive and expensive technique in such a great number of T2DM patients (10). Consequently, it is of benefit in identifying people suffering from NAFLD using simple methods, in order to detect those likely to progress to NASH or advanced liver disease (10). Nutrition is known as a main modifiable environmental factor in the development and management of NAFLD (11). A few researchers have clarified that antioxidant intake plays a crucial role in protecting against oxidative damage and relevant inflammatory complications in people with NAFLD (12). Currently, dietary total antioxidant capacity (DTAC) is regarded as a useful index to investigate the whole antioxidant capacity of foods (13). In comparison to a simple sum of certain dietary antioxidants, DTAC provides the cumulative capability of the total dietary antioxidants (13). Accumulating evidence suggests that DTAC is inversely associated with adverse health consequences such as cardiovascular diseases, T2DM, cancer deaths, and obesity (14–17). A recent case-control study declared that high DTAC is significantly related to decreased risk of NAFLD (12). Additionally, patients with greater DTAC indicated lower odds of NAFLD in comparison to lower amounts of DTAC (18).

Overall, the present study focused on the changes in blood glucose, lipid profile, transaminases, and IR among T2DM patients with and without NAFLD in order to present potential prognostic markers for identifying diabetic patients being at high risk of NAFLD. Furthermore, people with T2DM need to specifically be considered in the assessment of potential dietary prevention strategies for NAFLD. Nevertheless, to the best of the authors’ knowledge, the association between DTAC and the development of NAFLD in T2DM patients has not yet been investigated. Thus, the present study was conducted to evaluate probable association between DTAC and odds of NAFLD in T2DM patients.

## Materials and methods

### Study population

This cross-sectional study was conducted between April 2021 and February 2022 and was approved by the Ethics Committee of the Shahid Beheshti University of Medical Sciences (NO: IR.SBMU.NNFTRI.REC.1399.061). Eligible volunteers were selected by the use of consecutive-sampling method and provided with informed written consent, prior to study commencement.

### Inclusion criteria

The study population was recruited from people with T2DM referred to the diabetes clinic affiliated with the Institute of Diabetes and Metabolism, Iran University of Medical Sciences, Tehran, Iran. Participants in the NAFLD group (*n* = 133) were individuals aged between 18 and 70 years old with confirmed T2DM for over 2 years and CAP (Controlled attenuation parameter) score > 270 dB/m. The non-NAFLD group (*n* = 67) included individuals with 18–70 years of age and diagnosis of T2DM for over 2 years, as well as CAP score ≤ 270 dB/m. In addition, no participants in the NAFLD or non-NAFLD group was on insulin therapy. Body mass index (BMI) ≥ 23 kg/m^2^ was another inclusion criterion for participants in the both study groups. NAFLD was diagnosed using the findings of Fibroscan performed by an expert physician.

### Exclusion criteria

**FIGURE 1:**
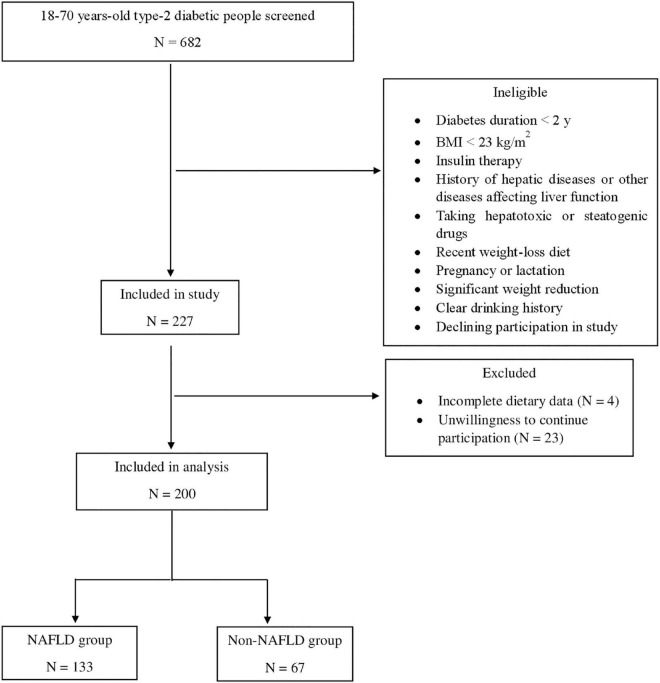
Flowchart of the study’s participants.

People with a history of any type of pathologically confirmed cancer, under chemotherapy or radiotherapy (due to cancer), drug use, chronic inflammatory disease, heart failure, myocardial infarction, and kidney disease were not included in the study. Moreover, Participants were excluded for recently weight-loss diet, taking weight-loss medications, pregnancy, lactation, more than 10% weight reduction during the last 6 months, history of hepatic diseases such as hepatitis, autoimmune disease, biliary disease, hereditary disorders of the liver including Wilson’s disease and hemochromatosis, and using toxins or drugs affecting the liver such as NSAIDs, anti-inflammatory drugs, etc. Participants with a clear drinking history (≥ 21 units/week in men and ≥ 14 units/week in women) were not included in the research. All of the studied patients reported that they do no drink alcohol at all. The flowchart of participants selection is shown in Figure 1.

### Overall, anthropometric, and physical activity evaluations

Required information about age, sex, smoking status, duration of diabetes, and use of supplements were collected *via* a standard questionnaire. In order to measure CAP score, transient elastography (TE) equipped with M and XL probes (Fibroscan^®^) was used. A digital scale (Seca, Germany) was used to evaluate individuals’ weight (kg), unshod and to the nearest 100 g. Height was measured using a tape measure and in a standing position to the nearest 0.5 cm. Finally, BMI was calculated by dividing weight (kg) by the square of height (meters). In order to determine the level of physical activity during the last 7 days, the International Physical Activity Questionnaire (IPAQ) short form was applied, and it was expressed as metabolic equivalent task (MET)-min/week (19). The validity and reliability of this questionnaire have been already examined in Iranian adult women. Blood pressure was measured on the basis of standard protocols using an automatic sphygmomanometer (OMRON, Mannheim, Germany).

### Laboratory tests

Venous blood samples were collected after 10–12 h of overnight fasting. Enzymatic colorimetric method was used to measure the levels of fasting blood glucose. Serum levels of Triglyceride (TG), Total Cholesterol (TC), and high-density lipoprotein (HDL) were calculated by the use of enzymatic assays and standard biochemical kits (Pars Azmun Co., Iran). Between- and within-run coefficient of variations were less than 6.2%. Low-density lipoprotein (LDL) was calculated by modified version of Friedewald equation (20). ECLIA method and Roche Diagnostics kits (Roche Cobas 6000 analyzer) were used to measure serum insulin. HOMA-IR (Homeostatic Model Assessment for Insulin Resistance) was calculated by the following equation: [*fastinginsulin*(μU/*mL*)×*fastingglucose*(*mmol*/L)]/22.5 (21). QUICKI (Quantitative Insulin Sensitivity Check Index) was computed as 1/[*log*(*fastinginsulin*[*cpsbreak*]*in*μU/*mL*)+*log* (*fastingglucoseinmg*/*dL*)] (22). TyG (Triglyceride and glucose) index was determined as *Ln*[*TG*(*mg*/*dL*)×fasting[*cpsbreak*]*glucose*(*mg*/*dL*)/2] (23).

### Dietary assessment and calculation of dietary total antioxidant capacity

Dietary intakes of participants during the last year were examined using a semi-quantitative food frequency questionnaire (FFQ) with 147 item which its reliability and validity in Iranian population have been previously evaluated (24). An expert dietitian, being totally unaware of the participants’ situation (regarding having NAFLD), administered the FFQ *via* face-to-face interviews. Subjects reported how often they consumed each food item during the last year and household measures were used to convert usual intakes to grams per day (25). Subsequently, Nutritionist 4 software (First Databank Inc., Hearst Corp., San Bruno, CA, USA) modified for Iranian foods determined the amount of total energy and dietary nutrients consumed every day. In the present study, DTAC was calculated *via* ferric reducing antioxidant power (FRAP). In order to obtain the total antioxidant capacity (TAC) (in mmol) of each food, published databases with antioxidant capacity of foods measured by the use of FRAP, were applied. When TAC of food items was unavailable, the amount of the closest comparable food was allocated (26). In order to calculate DTAC (in mmol), the daily intake of each food item over all products and units of antioxidant content (derived from an antioxidant index database) were summed (27).

### Statistical analysis

The normality of data was assessed using the Kolmogorov–Smirnov test and the histogram chart. By the use of the independent Student’s *t*-test and Mann–Whitney test, general characteristics with normal and abnormal distributions were compared between the study groups, respectively, and for qualitative variables, *X*^2^ test was applied. Next, subjects were categorized into tertiles of DTAC. General characteristics, biochemical parameters, and dietary intakes across tertiles of DTAC were evaluated by the use of Kruskal–Wallis test and analysis of covariance (ANCOVA) for continuous variables and *X*^2^ test for categorical variables.

Quantitative and qualitative variables were reported as mean ± SD (standard deviation) and percentages, respectively. To assess the association between biochemical factors and NAFLD in diabetic patients, binary logistic regression in different models was used. In addition, multiple logistic regression models were applied to determine unadjusted and adjusted ORs for DTAC. In all analyses, the first tertile of biochemical factors or DTAC was regarded as the reference category. A broad range of confounders was controlled to examine whether the association was independent of them. All the statistical analyses were conducted using SPSS (SPSS Inc., version 25). *P* values less than 0.05 were considered as significant.

## Results

Overall characteristics of individuals across the study groups are presented in Table 1. No statistically significant difference was found in age between the NAFLD and non-NAFLD groups. Among the diabetic patients with NAFLD and without NAFLD, 58.64 and 44.77 % were women, respectively. In terms of BMI, subjects in the NAFLD group significantly presented greater BMI compared with those in the non-NAFLD group. Besides, diabetic patients with NAFLD (NAFLD group) higher levels of TC, TG, LDL, transaminases, HbA1c, and TyG index, in comparison to those without NAFLD. Moreover, IR was more prevalent among them. The two groups, however, showed no significant differences in current smoking, diabetes duration, blood pressure, physical activity, FBS, HDL, and DTAC.

**TABLE 1:** Baseline characteristics of type 2 diabetic patients with or without non-alcoholic fatty liver disease (NAFLD)^†^.

Variables	NAFLD group (*n* = 133)	Non-NAFLD group (*n* = 67)	*P-value* ^‡^
Age (y)	52.19 ± 9.06	52.24 ± 9.75	0.84
Gender (female) (%)	58.64	44.77	0.07
Current smokers (%)	18.04	16.41	0.84
Diabetes duration (y)	8 ± 5.26	10 ± 6.77	0.27
Weight (kg)	81.4 ± 15.08	72.7 ± 10.91	< 0.001
BMI (kg/m^2^)	30.07 ± 4.06	26.17 ± 3.42	< 0.001
SBP (mmHg)	123 ± 14.55	125 ± 16.03	0.58
DBP (mmHg)	78 ± 10.42	75 ± 9.02	0.11
Physical activity (MET-min/week)	950.83 ± 1757.85	738.06 ± 683.27	0.37
FBS (mg/dL)	150.53 ± 59.22	148.79 ± 60.81	0.36
TC (mg/dL)	153.68 ± 51.75	132.52 ± 35.69	0.002
TG (mg/dL)	179.98 ± 173.01	141.69 ± 128.45	0.005
HDL (mg/dL) (*n* = 197)	49.53 ± 12.51	49.31 ± 13.53	0.6
LDL (mg/dL) (*n* = 197)	77.54 ± 27.77	66.37 ± 23.6	0.005
SGPT (IU/L)	20.05 ± 10.62	16.49 ± 7.28	0.02
SGOT (IU/L)	21.38 ± 9.05	18.82 ± 8.53	0.04
HbA1c (%) (*n* = 198)	7.92 ± 1.85	7.33 ± 1.64	0.01
Insulin (μU/mL) (*n* = 197)	8.77 ± 6.56	5.73 ± 3.36	< 0.001
QUICKI (*n* = 197)	0.33 ± 0.03	0.35 ± 0.02	< 0.001
HOMA–IR (*n* = 197)	3.23 ± 2.79	1.89 ± 0.94	< 0.001
TyG index	4.02 ± 0.31	3.91 ± 0.3	0.02
DTAC (mmol)	16.1 ± 10.48	15.11 ± 5.77	0.44

^†^Values are mean ± SD, unless indicated. ^‡^*P-values* were obtained from Mann Whitney *U* test or chi-square test, where appropriate unless QUICKI and TyG index for which they were obtained from independent sample test. BMI, Body mass index; SBP, systolic blood pressure; DBP, diastolic blood pressure; MET, metabolic equivalents; FBS, fasting blood glucose; TC, total cholesterol; TG, triglyceride; HDL, high-density lipoprotein-cholesterol; LDL, low-density lipoprotein-cholesterol; SGPT, serum glutamate pyruvate transaminase; SGOT, serum glutamic-oxaloacetic transaminase; HbA1c, hemoglobin A1c; QUICKI, quantitative insulin-sensitivity check index; HOMA-IR, Homeostatic Model Assessment of Insulin Resistance; TyG index, Triglyceride-glucose index; DTAC, dietary total antioxidant capacity.

Among categories of DTAC, a significant difference was found in terms of age, diastolic blood pressure (DBP), and insulin levels. However, BMI, physical activity, blood pressure, diabetes duration, and biochemical parameters did not differ significantly across categories of DTAC. We found that individuals in the top tertile had significantly higher intakes of monounsaturated fatty acid (MUFA), polyunsaturated fatty acid (PUFA), dietary fiber, fruits, vegetables, low-fat dairy products, and poultry. Additionally, the intake of whole grains, legumes, nuts, seeds, green/black tea, and coffee was significantly higher in them (Table 2).

**TABLE 2:** Characteristics, biochemical parameters, and dietary intakes of study participants across tertiles of dietary total antioxidant capacity^†^.

Variables	Dietary total antioxidant capacity^‡^			*P-value* ^§^
	T1	T2	T3	
Subjects, n	66	67	67	
Age (y)	52.05 ± 8.52	50.09 ± 10.05	54.48 ± 8.77	0.02
Gender (female) (%)	63.63	49.25	49.25	0.15
Current smokers (%)	12.12	26.86	13.43	0.04
Diabetes duration (y)	9 ± 5.43	8 ± 5.78	9.64 ± 6.17	0.13
SBP (mmHg)	125 ± 16.39	121 ± 11.66	124 ± 16.48	0.4
DBP (mmHg)	80 ± 10.7	75 ± 8.75	76 ± 9.86	0.01
Weight (kg)	76.72 ± 13.06	80.87 ± 17.29	77.86 ± 12.2	0.51
BMI (kg/m^2^)	28.71 ± 3.88	29.29 ± 4.93	28.29 ± 3.9	0.63
Physical activity (MET-min/week)	591.51 ± 497.34	1194.74 ± 2249.06	848.11 ± 1091.26	0.64
FBS (mg/dL)	153.58 ± 68.04	142.75 ± 52.05	153.58 ± 58.03	0.44
SGOT (IU/L)	20.7 ± 9.08	21.7 ± 10.05	19.16 ± 7.43	0.34
SGPT (IU/L)	19.7 ± 12.64	19.22 ± 8.61	17.66 ± 7.3	0.66
TC (mg/dL)	154.82 ± 62.17	140.85 ± 36.65	144.24 ± 40.94	0.32
TG (mg/dL)	171.67 ± 206.59	159.45 ± 100.46	170.42 ± 158.55	0.93
HDL (mg/dL)	50.29 ± 10.18	47 ± 13.45	51.09 ± 14.28	0.14
LDL (mg/dL)	78.41 ± 28.07	70 ± 26.56	72.89 ± 25.74	0.31
HbA1c (%)	7.77 ± 1.72	7.63 ± 1.81	7.76 ± 1.87	0.67
Insulin (μU/mL)	6.74 ± 4.18	9.81 ± 8.13	6.63 ± 3.6	0.01
HOMA-IR	2.54 ± 2.28	3.36 ± 3.05	2.42 ± 1.6	0.05
TyG index	3.99 ± 0.32	3.97 ± 0.28	3.99 ± 0.33	0.87
QUICKI	0.34 ± 0.03	0.33 ± 0.03	0.34 ± 0.03	0.07
**Dietary intakes**				
Carbohydrate (% of energy)	57.2 ± 8.61	56.73 ± 9.04	58.95 ± 10.44	0.26
Fat (% of energy)	31.95 ± 7.92	32.35 ± 11.16	31.15 ± 11.75	0.54
Protein (% of energy)	14.1 ± 2.56	18.67 ± 32.63	18.68 ± 34.99	0.25
SFA (% of energy)	10.25 ± 4.01	10.77 ± 6.64	9.7 ± 5.83	0.37
MUFA (g/d)	22.97 ± 10.54	30.03 ± 10.9	34.75 ± 18.88	< 0.001
PUFA (g/d)	14.3 ± 8.02	19.12 ± 7.26	22.66 ± 15.07	< 0.001
Dietary fiber (g/d)	31.53 ± 16.12	45.76 ± 21.82	48.77 ± 20.74	< 0.001
Whole grains (g/d)	98.39 ± 91.84	180.55 ± 154.94	173.9 ± 167.63	0.004
Low-fat dairy products (g/d)	158.27 ± 139.5	211.5 ± 204.88	278.33 ± 262.1	0.01
High-fat dairy products (g/d)	52.45 ± 111.43	67.62 ± 109.17	56.62 ± 104.96	0.07
Fish (g/d)	5.16 ± 8.41	5.87 ± 6.58	6.74 ± 9	0.24
Fruits (g/d)	355.78 ± 209.1	508.85 ± 302.39	773.31 ± 560.78	< 0.001
Vegetables (g/d)	349.63 ± 215.55	403.91 ± 225.24	489.04 ± 285.82	0.005
Green/black tea (g/d)	400.47 ± 247.29	724.41 ± 324.88	1449.62 ± 1159.82	< 0.001
Nuts (g/d)	6.23 ± 7.97	7.51 ± 9.12	10.83 ± 11.78	0.002
Legumes (g/d)	21.29 ± 24.28	31.22 ± 23.53	33.25 ± 47.27	0.003
Red meats (g/d)	14.27 ± 18.51	21.82 ± 19.18	19.85 ± 28.89	0.001
Organ meats (g/d)	1.85 ± 2.87	4.32 ± 8.58	3.92 ± 8.94	0.16
Poultry (g/d)	24.2 ± 19.01	37.2 ± 46.17	49.2 ± 55.78	< 0.001
Coffee (g/d)	16.73 ± 27.26	28.22 ± 63.4	100.59 ± 218.5	0.02
Sweets (g/d)	2.15 ± 3.58	2.41 ± 3.33	3.12 ± 7.27	0.09
Oil and olive oil (g/d)	3.87 ± 6.15	3.71 ± 5.96	6.79 ± 10.87	0.18
Seeds (g/d)	3.8 ± 8.04	6.29 ± 12.2	13.74 ± 36.05	0.03
Salt (g/d)	7.52 ± 7.25	4.12 ± 5.32	6.01 ± 5.77	0.001
Sugar-sweetened beverages (g/d)	17.36 ± 54.15	23.37 ± 63.5	15.19 ± 33.65	0.3

^†^Values are mean ± SD, unless indicated. ^‡^Individuals in the first tertile of DTAC had DTAC score less than 11.55; second tertile: between 11.55 and 16.68 and third tertile: more than 16.68. ^§^*P-values* were obtained from Kruskal-Wallis test or chi-square test, where appropriate unless QUICKI and TyG index for which they were obtained from one-way ANOVA. SBP, systolic blood pressure; DBP, diastolic blood pressure; BMI, Body mass index; MET, metabolic equivalents; FBS, fasting blood glucose; SGOT, serum glutamic-oxaloacetic transaminase; SGPT, serum glutamate pyruvate transaminase; TC, total cholesterol; TG, triglyceride; HDL, high-density lipoprotein-cholesterol; LDL, low-density lipoprotein-cholesterol; HbA1c, hemoglobin A1c; HOMA-IR, Homeostatic Model Assessment of Insulin Resistance; TyG index, Triglyceride-glucose index; QUICKI, quantitative insulin-sensitivity check index; SFA, saturated fatty acid; MUFA, monounsaturated fatty acid; PUFA, polyunsaturated fatty acid.

Multiple logistic regression analysis was applied to examine whether certain biochemical factors were independently and significantly associated with the presence of NAFLD in people with diabetes (Table 3). For all T2DM patients, LDL-C, HbA1c, HOMA-IR, and TyG index were significantly associated with greater odds of having NAFLD, according to the fully adjusted model. On the other hand, the analysis showed that high QUICKI was a protective factor for NAFLD. Specifically, those with LDL (OR = 3.08, 95% CI: 1.16–8.16, *P* = 0.02) levels in tertile 3 had significantly increased odds for NAFLD, independently of age, gender, diabetes duration, smoking status, physical activity, BMI, waist circumference, HOMA-IR, HbA1c, and energy. Additionally, compared to tertile 1, HbA1c levels in tertile 3 were independently associated with a significantly increased odd for NAFLD (OR = 2.92, 95% CI: 1.14–7.45, *P* = 0.02). Similarly, T2DM patients with higher amounts of HOMA-IR (> 2.7) had significantly 5.33 times the odds (OR = 5.33, 95% CI: 1.83–15.5, *P* = 0.002) of NAFLD compared to T2DM patients with lower amounts (< 1.62) after controlling for the potential confounders. Also, diabetic patients with higher levels of QUICKI had reduced OR in NAFLD than those with lower levels (OR = 0.18, 95% CI: 0.06–0.54, *P* = 0.002). Another parameter independently associated with an increased odds of having NAFLD was TyG index (OR: 2.99, 95% CI: 1.22–7.32; *P* = 0.01).

**TABLE 3:** Crude and multivariable-adjusted odds ratios (ORs) and 95% confidence intervals (95% CIs) for non-alcoholic fatty liver disease (NAFLD) in participants with type 2 diabetes across tertiles of biochemical parameters.

Model	Odds ratio (95% CI)			*P*-trend^†^
	T1	T2	T3	
		FBS, mg/dL		
	< 121	121–149.98	149.98 <	
Subjects, n	68	65	67	
Model 1	1 (Ref)	1.97 (0.94–4.11)	1.43 (0.7–2.89)	0.3
Model 2	1 (Ref)	2.45 (1.02–5.85)	1.9 (0.77–4.71)	0.13
		TC, mg/dL		
	< 124.98	124.98–156	156 <	
Subjects, n	66	68	66	
Model 1	1 (Ref)	1.19 (0.59–2.37)	3 (1.37–6.54)	0.006
Model 2	1 (Ref)	0.83 (0.34–2.04)	3.18 (1.22–8.26)	0.02
Model 3	1 (Ref)	0.75 (0.29–1.9)	2.26 (0.82–6.2)	0.13
		TG, mg/dL		
	< 102	102–162.96	162.96 <	
Subjects, n	68	65	67	
Model 1	1 (Ref)	2.02 (0.99–4.15)	2.46 (1.18–5.1)	0.01
Model 2	1 (Ref)	2.06 (0.89–4.77)	2.01 (0.85–4.73)	0.08
Model 3	1 (Ref)	2.06 (0.84–5)	1.8 (0.72–4.48)	0.16
		HDL-C, mg/dL		
	< 42.99	42.99–54	54 <	
Subjects, n	65	69	63	
Model 1	1 (Ref)	1.53 (0.74–3.15)	1.16 (0.56–2.39)	0.66
Model 2	1 (Ref)	1.78 (0.72–4.37)	1.02 (0.4–2.61)	0.97
Model 3	1 (Ref)	1.91 (0.73–4.97)	1.31 (0.48–3.53)	0.59
		LDL-C, mg/dL		
	< 60	60–80	80 <	
Subjects, n	67	65	65	
Model 1	1 (Ref)	1.57 (0.78–3.16)	3.44 (1.58–7.47)	0.002
Model 2	1 (Ref)	1.28 (0.54–3.03)	4.03 (1.58–10.29)	0.004
Model 3	1 (Ref)	1.07 (0.43–2.63)	3.08 (1.16–8.16)	0.02
		SGOT, IU/L		
	< 16	16–22	22 <	
Subjects, n	72	58	70	
Model 1	1 (Ref)	0.86 (0.42–1.75)	1.63 (0.79–3.35)	0.19
Model 2	1 (Ref)	0.57 (0.23–1.39)	1.64 (0.7–3.83)	0.25
Model 3	1 (Ref)	0.58 (0.22–1.48)	1.46 (0.59–3.6)	0.43
		SGPT, IU/L		
	< 13	13–20	20 <	
Subjects, n	67	61	72	
Model 1	1 (Ref)	0.97 (0.48–1.99)	2.05 (0.98–4.27)	0.05
Model 2	1 (Ref)	1.33 (0.57–3.11)	2.38 (0.97–5.81)	0.06
Model 3	1 (Ref)	1.36 (0.55–3.37)	1.91 (0.75–4.88)	0.24
		HbA1c, %		
	< 6.7	6.7–8	8 <	
Subjects, n	65	66	68	
Model 1	1 (Ref)	1.54 (0.76–3.13)	2.92 (1.37–6.23)	0.34
Model 2	1 (Ref)	1.47 (0.63–3.45)	2.92 (1.14–7.45)	0.02
		Fasting insulin, μU/mL		
	< 4.59	4.59–8.39	8.39 <	
Subjects, n	66	66	66	
Model 1	1 (Ref)	2.07 (1.02–4.22)	3.95 (1.81–8.6)	< 0.001
Model 2	1 (Ref)	1.57 (0.67–3.68)	2.2 (0.86–5.64)	0.09
		HOMA-IR		
	< 1.62	1.62–2.7	2.7 <	
Subjects, n	66	66	66	
Model 1	1 (Ref)	0.17 (0.7–0.4)	0.26 (0.11–0.62)	< 0.001
Model 2	1 (Ref)	1 (0.43–2.31)	5.33 (1.83–15.5)	0.004
		TyG index		
	< 3.83	3.83–4.06	4.06 <	
Subjects, n	66	67	67	
Model 1	1 (Ref)	1.95 (0.96–3.99)	2.45 (1.17–5.1)	0.01
Model 2	1 (Ref)	1.99 (0.86–4.63)	2.99 (1.22–7.32)	0.01
		QUICKI		
	< 0.32	0.32–0.35	0.35 <	
Subjects, n	66	66	66	
Model 1	1 (Ref)	0.26 (0.11–0.62)	0.17 (0.07–0.4)	< 0.001
Model 2	1 (Ref)	0.18 (0.06–0.55)	0.18 (0.06–0.54)	0.004

Model 1: Crude. Model 2: Adjusted for age (continuous), sex (male/female), physical activity (continuous), diabetes duration (continuous), current smoking (yes/no), BMI (continuous), waist circumference (continuous), and energy intake (kcal/d). Model 3: This model was additionally adjusted for HOMA-IR (continuous) and HbA1c (continuous). ^†^Binary logistic regression models were employed to obtain odds ratios (ORs) and 95% CIs. The overall trend of ORs was examined by the use of tertiles of the biochemical parameters as an ordinal variable in the model.

Multivariable-adjusted odds ratios and 95% CIs for NAFLD by tertiles of DTAC are indicated in Table 4. In the crude model, DTAC was not significantly associated with the odds of NAFLD in people with diabetes. However, after adjustment for potential confounders including age, gender, diabetes duration, smoking status, physical activity, BMI, waist circumference, and energy, the lowest adjusted OR was observed for the last tertile vs. the first one (OR: 0.28, 95% CI: 0.09–0.81, *P* = 0.02), meaning that diabetic patients in the highest tertile of DTAC had 72 % decreased risk of NAFLD compared with those in the lowest tertile. The association remained significant after additional adjustment for HOMA-IR, HbA1c, TG, and LDL levels (OR: 0.29, 95% CI: 0.09–0.93, *P* = 0.03). Importantly, a dose-response pattern was demonstrated for DTAC and risk of NAFLD (*P* = 0.04).

**TABLE 4:** Crude and multivariable-adjusted odds ratios (ORs) and 95% confidence intervals (95% CIs) for non-alcoholic fatty liver disease (NAFLD) in type 2 diabetes patients across tertiles of dietary total antioxidant capacity.

	Dietary total antioxidant capacity			*P*-trend^†^
	T1 (*n* = 66) < 11.55	T2 (*n* = 67) 11.55–16.68	T3 (*n* = 67) 16.68 <	
	OR	OR (95 % CI)	OR (95 % CI)	
Model 1	1 (Ref)	0.66 (0.31–1.4)	0.51 (0.24–1.07)	0.07
Model 2	1 (Ref)	0.38 (0.14–0.99)	0.28 (0.09–0.81)	0.02
Model 3	1 (Ref)	0.41 (0.14–1.15)	0.29 (0.09–0.93)	0.04

Model 1: Crude. Model 2: Adjusted for age (continuous), sex (male/female), physical activity (continuous), diabetes duration (continuous), current smoking (yes/no), BMI (continuous), waist circumference (continuous), and energy intake (kcal/d). Model 3: Further adjustments were made for HOMA-IR (continuous), HbA1c (continuous), triglycerides (continuous), and LDL (continuous) levels. ^†^Binary logistic regression models were employed to obtain odds ratios (ORs) and 95% CIs. The overall trend of ORs was examined by the use of tertiles of the DTAC as an ordinal variable in the model.

## Discussion

The present cross-sectional study investigated the associations between DTAC and NAFLD in people with diabetes. According to our results, patients in the NAFLD group (diabetes with NAFLD) had a higher BMI and serum levels of TC, TG, LDL, transaminases and HbA1C than the non-NAFLD group (diabetes without NAFLD). These patients also showed higher degrees of IR. The risk of NAFLD was significantly higher in the third tertile of LDL and HbA1c compared to the first tertile. The risk of NAFLD also increased significantly with increasing IR and decreasing insulin sensitivity. After fully adjustment for age, sex, physical activity, diabetes duration, current smoking, BMI, waist circumference, energy intake, Triglycerides, LDL, HbA1c, and HOMA-IR, risk of NAFLD was significantly lower in patients with higher DTAC. However, no significant difference was observed in DTAC score between the study groups. To the best of our knowledge, this is the first study reporting the inverse associations between DTAC and odds of NAFLD in people with diabetes.

Dietary total antioxidant capacity (DTAC) is a measure of the antioxidant capacity of the diet and is considered a good indicator of diet quality (28). An inverse association has been reported between DTAC and numerous chronic diseases including cardiovascular diseases (29), diabetes (16, 30), metabolic syndrome (31) and NAFLD (32, 33). Similar metabolic abnormalities are common among these disorders, which are exacerbated by combination of diabetes and NAFLD. Previously, an inverse association of antioxidant-rich dietary patterns, including the Dietary Approaches to Stop Hypertension (DASH) diet (34–36), plant-based diet (37, 38), with the risk of NAFLD and diabetes has been reported separately. All of these diets are rich in antioxidant compounds and therefore have antioxidant effects.

Oxidative stress is involved in the pathogenesis of NAFLD by promoting IR, dyslipidemia (39). Antioxidant-rich foods/diets might be effective in reinforcing antioxidant defense by reducing lipid peroxidation, cellular and organ damage, and insulin sensitivity (40). Increasing the antioxidant capacity of cells improves glucose and lipid metabolism and reduces the risk of NAFLD (39, 40).

The findings of this study showed that the risk of NAFLD in people with diabetes increased significantly with increasing IR. Study groups were significantly different in terms of insulin resistance and sensitivity, although this difference was not found among DTAC tertiles. IR is a mediator in the effect of oxidative stress on lipid profile (41). Another factor that may play a role in DTAC and NAFLD risk reduction is fiber, which contributes to weight loss, improved insulin sensitivity, dyslipidemia, and glycemic control (42).

Although dietary antioxidants play a role in reducing obesity and related disorders by inhibiting fat absorption, stimulating adipose tissue catabolism, inhibiting the proliferation and differentiation of adipocytes (43), no significant difference in weight was observed between the three tertiles of DTAC. However, it has been suggested that the association of DTAC with cardiovascular risk factors is independent of weight (44).

In this study, no difference was observed between DTAC between NAFLD and non-NAFLD groups, which is contrary to previous findings in diabetic patients (45) and NAFLD patients (33). However, in these studies, the comparisons were made between patients and healthy individuals, while in our study, diabetic people with and without NAFLD were compared. Oxidative stress reduces insulin secretion by damaging the mitochondria of pancreatic beta cells and causes diabetes (46, 47).

The present study has several limitations. First, due to the cross-sectional nature of the study design, cause and effect relationships could not be confirmed. The use of United States Department of Agriculture (USDA) food composition table was another limitation of the study due to incomplete database for the content of Iranian food antioxidant. The validity of DTAC obtained from the FFQ in the Iranian diet has not been examined previously. Also, the use of FFQ is associated with recall bias.

In conclusion, higher DTAC was associated with a decreased odds of having NAFLD in people with diabetes.

## Data availability statement

The raw data supporting the conclusions of this article will be made available by the authors, without undue reservation.

## Ethics statement

The studies involving human participants were reviewed and approved by Shahid Beheshti University of Medical Sciences, Tehran, Iran. The patients/participants provided their written informed consent to participate in this study.

## Author contributions

MS conceived and designed the study, performed the statistical analysis, interpreted the data, and drafted the manuscript. SS and AM contributed to the sampling selection schedule. ZY and MSh drafted the manuscript. HP contributed to the data interpretation. MK, FA-S, AH, and MSh made critical revisions to the manuscript. MK and AH contributed to the study supervision. All authors have read and approved the final version to be published.
